# Automated parcellation of the brain surface generated from magnetic resonance images

**DOI:** 10.3389/fninf.2013.00023

**Published:** 2013-10-22

**Authors:** Wen Li, Nancy C. Andreasen, Peg Nopoulos, Vincent A. Magnotta

**Affiliations:** ^1^Department of Biomedical Engineering, The University of IowaIowa City, IA, USA; ^2^Department of Radiology, The University of IowaIowa City, IA, USA; ^3^Department of Psychiatry, The University of Iowa Roy and Lucille Carver College of MedicineIowa City, IA, USA

**Keywords:** cerebral cortex, cortical parcellation, surface registration, spherical demons, cytoarchitecture, magnetic resonance imaging

## Abstract

We have developed a fast and reliable pipeline to automatically parcellate the cortical surface into sub-regions. The pipeline can be used to study brain changes associated with psychiatric and neurological disorders. First, a genus zero cortical surface for one hemisphere is generated from the magnetic resonance images at the parametric boundary of the white matter and the gray matter. Second, a hemisphere-specific surface atlas is registered to the cortical surface using geometry features mapped in the spherical domain. The deformation field is used to warp statistic labels from the atlas to the subject surface. The Dice index of the labeled surface area is used to evaluate the similarity between the automated labels with the manual labels on the subject. The average Dice across 24 regions on 14 testing subjects is 0.86. Alternative evaluations have also chosen to show the accuracy and flexibility of the present method. The point-wise accuracy of 14 testing subjects is above 86% in average. The experiment shows that the present method is highly consistent with FreeSurfer (>99% of the surface area), using the same set of labels.

## 1. Introduction

Anatomical magnetic resonance (MR) imaging provides the ability to obtain quantitative measurements of brain structures. These measurements can be used to study the neurobiology of various diseases. The resulting quantitative measurements can be used to study subtle morphological changes if the methods are sufficiently reliable. The analysis of anatomical MR images has evolved from the tissue classification (labeling of voxels into constituent components of gray matter, white matter, and CSF) to labeling of anatomical regions of interest. The definition of anatomical regions of interest began with subcortical structures (e.g., caudate and putamen) because of their relative well- defined borders with surrounding structures such as white matter and ventricular CSF. With the development of three-dimensional (3D) MR sequences, segmentation of additional anatomical structures such as the hippocampus, amygdala, and globus pallidus has became possible. The segmentation of the cerebral cortex has been significantly more challenging. The human cerebral cortex is a highly convoluted structure with significant anatomical variability and heterogeneity across individuals (Uylings et al., 2005). While the surface of the cortex can be readily generated from MR images, the automated labeling remains a challenging task.

The cerebral cortex can be divided into distinct regions based upon cytoarchitecture (Brodmann, 2006) function (Roland and Zilles, 1998), or cortical features. While divisions based on cytoarchitecture are possible using post-mortem brains, it is not currently possible to define the cortical layers from *in vivo* data collected on 1.5 or 3T MR scanners. It may be possible to collect data at 7T that reflects the cytoarchitecture of the cortex (Zwanenburg et al., 2012). While the neuroimaging community has significant interest in such approaches, relatively few anatomical imaging studies to date have been conducted at 7T. In the absence of cytoarchitecture information, parcellation schemes applied to *in vivo* data have defined functional distinct regions based on sulcal boundaries. Such parcellation schemes allow anatomical images to be segmented without the acquisition of functional data, and allow such schemes to be applied to the large number of retrospective imaging data that has been acquired.

A number of groups have defined functionally relevant regions of the cortex using cortical features as the boundaries between regions. (Caviness et al., 1996) and (Rademacher et al., 1992) defined a parcellation scheme that divided the cortex into forty-eight regions based on 16 coronal planes and 31 major sulci. (Desikan et al., 2006) divided the brain into 34 cortical regions of interest per hemisphere. In this study, curvature based information was used to guide the parcellation of the brain on an inflated representation of the cortex. More recently, a refined parcellation scheme was developed by (Destrieux et al., 2010), which divides the cortical surface into 74 sulcal and gyral regions of interest. Our group has also developed a parcellation scheme of the cerebral cortex that utilized the cortical surface, MR images, and anatomical landmarks to generate 24 regions of interest per hemisphere. The reliability of this parcellation scheme was evaluated between expert anatomical raters (Crespo-Facorro et al., 2000a; Kim et al., 2003). In addition, the same method was applied to 25 patients with schizophrenia and 25 normal controls. The labeling of the cortex took a significant duration of time. We have estimated that it typically took approximately 24 h of human rater time to complete the manual labeling of the cerebral cortex using the guidelines that were developed. With such time-intensive techniques, it is clear that the manual parcellation of the cerebral cortex can only be applied to relatively small samples, suffers from rater bias and drift, and requires a significant time investment to train raters.

Several large imaging studies are currently being conducted to study a variety of neurological and psychiatric disorders (e.g., Goldman et al., 2008; Nopoulos et al., 2010; Trzesniak et al., 2012). These studies would benefit from the ability to generate quantitative measurements of the cerebral cortex. Many of these studies are collecting thousands of MR scans. Therefore, it is not practical to apply manual methods to these studies. To study cortical morphology and the changes associated with disease, automated algorithms are required. A number of semi-automated and automated procedures have been proposed in the literature. Such methods typically employ registration and/or feature extraction to bring an atlas into correspondence with the new dataset. The new dataset is then labeled by either directly mapping the anatomical labels from the atlas onto the subject or by mapping probabilistic information from the atlas into subject space and then applying classifiers (statistical or artificial intelligence based) to generate a labeling of the subject data. Below we summarize some of the methods that have been employed to date to automate the labeling of the cerebral cortex. This is not intended to be a comprehensive overview, but to provide context for the work proposed in this application.

Image based registration is one of first methods that was employed for automated labeling of the cerebral cortex. Collins et al. employed two methods to drive the registration using their Automatic Nonlinear Image Matching and Anatomical Labeling (ANIMAL) algorithm. This algorithm is initialized with a linear registration. In the non-linear portion of the algorithm, a hierarchical registration is used to refine an estimate of a local deformation vector at each grid node. In the first approach, the ANIMAL algorithm is coupled with sulcal constraints that are extracted from the MR images. The sulcal constraints were shown to improve the correspondence by more than 50% as compared to image registration alone (Collins et al., 1998). Collins et al. also combined the ANIMAL registration with tissue classification (INSECT) to enhance the labeling of the cortical surface. The tissue classification information is coupled with the maximum probability atlas to label cortical and subcortical regions of interest. The Kappa index for the resulting segmentations was 0.657 (Collins et al., 1999). In both of these approaches the gray matter ribbon was labeled. Other groups have also integrated cortical features within various image registration algorithms (Liu et al., 2004; Joshi et al., 2007a; Postelnicu et al., 2009; Auzias et al., 2011). All of these studies show that incorporation of cortical features into a volumetric based image registration algorithm substantially improves the registration of the cortical surface. Due to the large heterogeneity in the cortical surface across subjects, the choice of the atlas is an important consideration. (Heckemann et al., 2010) utilized a combination of multiple atlases and tissue class information to label the cortical surface. The labels in this study were volumetric and were compared against a manual rater. The resulting overlaps were reported as Dice and Jaccard metrics. The resulting Jaccard metric ranged from 0.33 to 0.93 with a mean of 0.69. (Sabuncu et al., 2010) improved Heckemann's method by proposing a probabilistic model to perform decision fusion of labels transferred from multiple atlases.

Techniques based on surface registration have become the most widely used approaches to automated cortical labeling. These techniques utilize sulcal and gyral information on the reconstructed surface as anatomical features, which are used to drive the registration. Surface based registration has a number of advantages over image registration for alignment of the cortical surface. First, the registration problem can be simplified from 3D to two-dimensions (2D) since the cortical surface can be represented as a 2D manifold. Second, the sulci are typically used to define boundaries between cortical regions of interest. They are easier to represent on the cortical surface than on 3D image, and topographic features such as curvatures can be readily calculated from the cortical surface (Schaer et al., 2008). Third, the average atlas generated from image registration tends to blur gyral and sulcal features as compared to surface based registration (Van Essen et al., 1998). In addition, the blurring tends to take place across features vs. along the cortical surface. Fourth, due to the highly folded structure of the cerebral cortex, it is difficult to generate measurements along the cortical surface using a 3D volume alone (Fischl et al., 1999). Some of the surface based methods require the surface features to be labeled prior to the surface registration step (Bookstein, 1991; Van Essen, 2005; Joshi et al., 2007b), while others utilize the whole surface and anatomical features (Fischl et al., 2004; Desikan et al., 2006; Yeo et al., 2010).

FreeSurfer is the most commonly utilized tool to perform automated labeling and utilizes surface registration to align the subject surface and atlas before using a non-stationary anisotropic Markov random field (MRF) to provide the anatomical labeling of the surface (Fischl et al., 2004). FreeSurfer has been shown to have good reliability. (Desikan et al., 2006) utilized the intraclass correlation coefficients (ICCs) to compare volumes of manually and automatically labeled regions of interests. The ICCs for 32 regions were from 0.62 to 0.98 with a mean of 0.84. The one limitation of this software is the significant computational resources that are required to run the tool. Based on our experience, the computational time can be up to 20 h per dataset to run the complete pipeline, which includes image alignment, tissue classification, surface generation, topology correction, and automated labeling. (Yeo et al., 2010) recently proposed a fast and landmark-free surface registration method. It was applied in the automated cortical surface parcellation using MR scans of 39 subjects. Thirty-six regions on each cortical surface were manually labeled by a neuroanatomist. A multi-resolution spherical diffeomorphic demons surface registration was performed to align cortical surfaces. The method was shown to be faster and achieved significantly higher overlap (Dice metric) between manually and automatically labeled regions.

Other approaches have been proposed for labeling of the cerebral cortex. (Klein et al., 2005) has developed an algorithm called Mindboggle. This algorithm utilizes linearly co-registered MR scans and extracts sulcal pieces. The sulcal pieces are then matched with a combination of atlas pieces to minimize a cost function. The resulting deformation is then used to warp the atlas labels onto the subjects. The sulcal pieces do not need to be manually labeled and are only used to bring two surfaces into registration.

We propose a fast fully automated method to parcellate the cortical surface. This method integrates with the BRAINS AutoWorkup procedure such that the entire process from raw scan to labeled surface is automated. The algorithm extends the prior work developed by (Yeo et al., 2010). The reliability of the method is compared to manual parcellation as well as to FreeSurfer using the same datasets.

## 2. Materials and methods

### 2.1. Data acquisition

The subjects in this study were enrolled voluntarily into a MR imaging protocol after informed written consent was obtained in accordance with the institutional review board at the University of Iowa. Fifty subjects were enrolled into a MR imaging study of schizophrenia, including 25 first episode patients (age: 19–39 years old, mean = 25.2) and 25 matched control subjects (age: 12–41 years old, mean = 25.6). Subjects were imaged using a multi-modal MR imaging protocol consisting of T1, T2, and proton density scans. The images were obtained on a GE Signa 1.5T MR scanner. The T1- weighted scans were acquired using a 3D spoiled recalled gradient echo sequence with the following scan parameters: *TE* = 5 ms, *TR* = 24 ms, Flip angle = 40 °, NEX = 2, FOV = 26 × 19.2 × 18.6 cm, Matrix = 256 × 256 × 192. The proton density and T2- weighted scans were acquired using a dual-echo fast spin-echo sequence with the following parameters: *TE* = 28/96 ms, *TR* = 3000 ms, slice thickness / gap = 3.0 mm / 0.0 mm, NEX = 1, FOV = 26 × 19 cm, Matrix = 256 × 192, ETL = 8.

One subject had an incomplete manual parcellation and was excluded from further study. The remaining forty-nine subjects were divided into two groups: a training set and a testing set. The subjects in the training set were used to develop hemisphere specific cortical surface atlases as described below. To generate a population atlas that represents a wide age range, 35 subjects were selected for the training set. The training set consisted of 16 patients with schizophrenia and nineteen healthy controls. The subjects had an age range of 12–41 years old with a mean of 25.6 years. The remaining fourteen subjects were used as the testing set for the developed automated cortical parcellation algorithm. This set of subjects varied in age from 16 to 39 years with a mean 24.9 years. The sample included eight subjects with schizophrenia and six control subjects.

### 2.2. Image pre-processing

For this study, only the T1- and T2-weighted scans were used in the analysis. The images were analyzed using an updated version of the BRAINS AutoWorkup pipeline (Pierson et al., 2011). This pipeline links together a number of stand-along applications built upon the ITK library linked together through the TCL scripting language. This is a fully automated procedure to analyze structural MR images that includes AC-PC alignment (BRAINSConstellationDetector), image co-registration (BRAINSFit), bias field correction / signal intensity normalization / tissue classification / brain extraction (BRAINSABC), and neural network anatomical labeling of the caudate, putamen, thalamus, globus pallidus, hippocampus, amygdala, and nucleus accumbens (BRAINSCut) (Kim and Johnson, 2010). The resulting continuous tissue classified image, binary image representing the brain, and the neural network defined regions for the caudate, putamen and thalamus were used to generate the cortical surface using a pipeline combining the ITK version 4 libraries and VTK as described below.

### 2.3. Cortical surface generation

The cortical surface generation involved several steps including topology correction, surface generation, surface decimation, surface smoothing, and generation of cortical features. We have previously reported on the methods employed for cortical surface generation (Li et al., 2011). A brief summary is provided here. To separate the cortical hemispheres and remove the brainstem and cerebellum, a simulated T1 weighted image generated from the BrainWeb Simulated Brain Database was created (Cocosco et al., 1997). An expert rater manually defined the ventricles, right and left hemispheres as well as the cerebrum, brainstem, and cerebellum on the BrainWeb T1 weighted image. The BrainWeb anatomical T1 weighted image was co-registered with each of the subjects AC-PC aligned anatomical T1 weighted image using a diffeomorphic demons registration (Vercauteren et al., 2009). The resulting deformation field was applied to BrainWeb based representations of the following structures: right hemisphere, left hemisphere, ventricles, brainstem, and cerebellum. The union of the warped ventricle label and the neural network defined subcortical regions (caudate, putamen, and thalamus) was calculated. Voxels on the continuous tissue classified image that overlap with the results from the union operation calculated in the previous step were assigned an image intensity value corresponding to pure white matter. This prevented the surface from entering the ventricles and eliminated the possibility that the surface could jump from the insula to the putamen. The brainstem and cerebellar regions were then used to remove these structures from the image by replacing the voxels that overlap these structures with a value of zero. The left and right hemisphere definitions were then used to limit the portion of the image considered during surface generation. This produced a separate surface for each hemisphere.

The cortical surface for each hemisphere was generated at the boundary between gray matter and white matter on the tissue classified image (i.e., greater than or equal to 50% white matter on the continuous tissue classified image). This avoids the problem of the “buried cortex" and resulted in a surface that could readily be corrected for topological defects to produce a genus zero surface. The topological defects were removed by first binary thresholding the tissue classified image and then performing topological correction on the binary image [see (Li et al., 2011) for details]. After topology correction was performed, the cortical surface was generated and decimated. Incremental edge collapse mesh decimation was applied to remove unnecessary number of vertices and triangles on the surface (Gelas et al., 2008). The decimation stops when the specified number of triangles remained on the resulting surface. In this pipeline, the number of triangles was reduced from approximately 250,000 to 70,000 after the decimation. The decimated surface was then smoothed using five iterations of Laplacian smoothing with a relaxation factor of 0.1.

**Figure 1:**
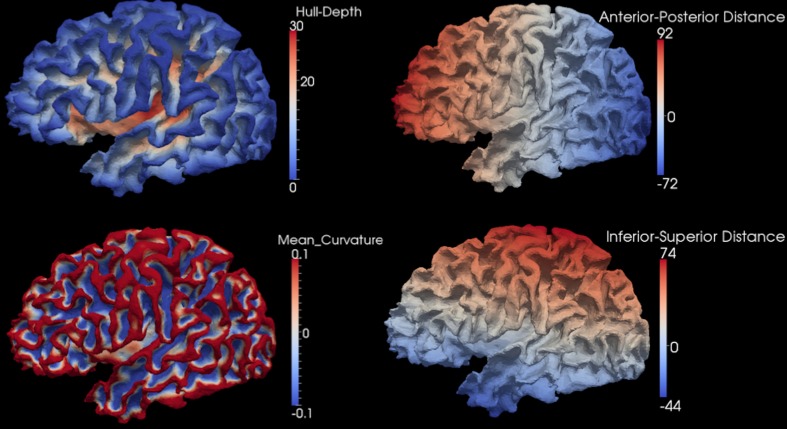
**Four geometry features calculated for the surface of one cortical hemisphere for a single subject.** Surfaces are colored by scalar values associated with vertices. Features names and scalar ranges are shown on the right side of each surface. Units: *Inferior-Superior Distance*, *Anterior-Posterior Distance*, and *Hull-Depth* are in millimeters; *Mean-Curvature* has the units of 1/millimeter.

After the smooth cortical surface was generated, the geometry features of the surface were calculated and associated with each vertex. Four geometry features were used in the automated parcellation method (Figure 1). The definitions for each geometry feature are as follows:

- Inferior-Superior Distance (*IS-Distance*): The inferior-superior distance is measured from each vertex point on the surface to the AC-PC line. The locations of the anterior commissure (AC) and posterior commissure (PC) were automatically estimated by the AutoWorkup pipeline. The feature helps to identify the location of the temporal pole (largest negative value) and the superior aspect of the central sulcus (largest positive value).
- Anterior-Posterior Distance (*AP-Distance*): The anterior-posterior distance is measured from each vertex point on the surface to the PC point. The feature helps to identify the frontal pole (largest negative value) and the occipital pole (largest positive value), as well as the central sulcus (approximately zero).
- *Hull-Depth*: The Euclidean distance in millimeters is measured from each vertex point to the closest point on a convex hull enclosing the cortical surface. The feature helps to identify deep grooves such as the insula as well as major sulci.
- *Mean-Curvature*: The mean curvature is calculated at each vertex point. It helps to identify secondary sulci and gyri.

### 2.4. Spherical mapping of the cortical surface

After the genus zero cortical surface was generated, the vertices and triangles on the surface were then mapped onto a sphere using surface parameterization (Gelas and Gouaillard, 2007; Li et al., 2011). The mapping is performed as follows: (1) split the genus zero surface into two half surfaces with a shared boundary; (2) generate a smooth boundary between the half surfaces; (3) map each of the half surfaces onto a unit disk with a fixed boundary; (4) project each disk onto a hemisphere using inverse stereo projection; 5) connect the two hemispheres to form a sphere. This mapping from the cortical surface onto the spherical domain provides a stable projection without significant dependencies on the selection of the polar points. Since the parameterization generates a one-to-one mapping, the geometry features calculated above can be readily projected onto the sphere and associated with the corresponding vertices.

### 2.5. Cortical surface registration

After mapping the cortical hemisphere onto the sphere, a multi-resolution spherical diffeomorphic demons registration (Yeo et al., 2010) was used to align the subject and atlas surfaces in the spherical domain. The multi-resolution deformable registration used different geometry features at each resolution level. The features were used in ascending order of geometry detail, with IS-Distance and AP-Distance having the coarsest geometry detail, Mean-Curvature having the finest, and Hull-Depth in the middle (Figure 1). To ensure sufficient movement of the surface vertices, the sphere was resampled onto a uniform icosahedral mesh. The mesh refinement (IC4, IC5, IC6, IC7) and surface features used at each level of the multi-resolution registration are summarized in Table 1. The subject's geometry features were smoothed and normalized on the icosahedral meshes before registration. The smoothing was applied by calculating the weighted average scalar values of the center vertex and its first order neighbor vertices (Yeo et al., 2010). Different weights were given to the center vertex vs. neighbor vertices. Parameter λ was used to control the weights, and the larger the value of λ the greater the amount of smoothing. Since surface features in levels (IC4, IC5, and IC6) are intrinsically smooth while level IC7 is more noisy, a relatively small λ = 0.5 was used for the former levels while λ = 1.0 was used for IC7. Given the variation in the geometric information and dynamic range of each scalar, different normalization procedures (piecewise rescaling, histogram matching, and clamping) were employed for the various scalar measures (Table 1). After the normalization, the normalized scalars had the following range: (1) *IS-Distance* and *AP-Distance* were rescaled between −1 and 1; (2) *Hull-Depth* histogram was matched to the target surface; and (3) *Mean-Curvature* was clamped between −1 and 1.

**Table 1:** **Surfaces and features used for multi-resolution registration**.

**Registration level**	**Icosahedral**	**Number of vertices**	**Feature**	**Normalization**
1	IC4	2,562	IS-Distance	Smoothing + Piecewise rescaling
2	IC5	10,242	AP-Distance	Smoothing + Piecewise rescaling
3	IC6	40,962	Hull-Depth	Smoothing + Histogram matching
4	IC7	163,842	Mean-Curvature	Smoothing + Clamping

**Figure 2:**
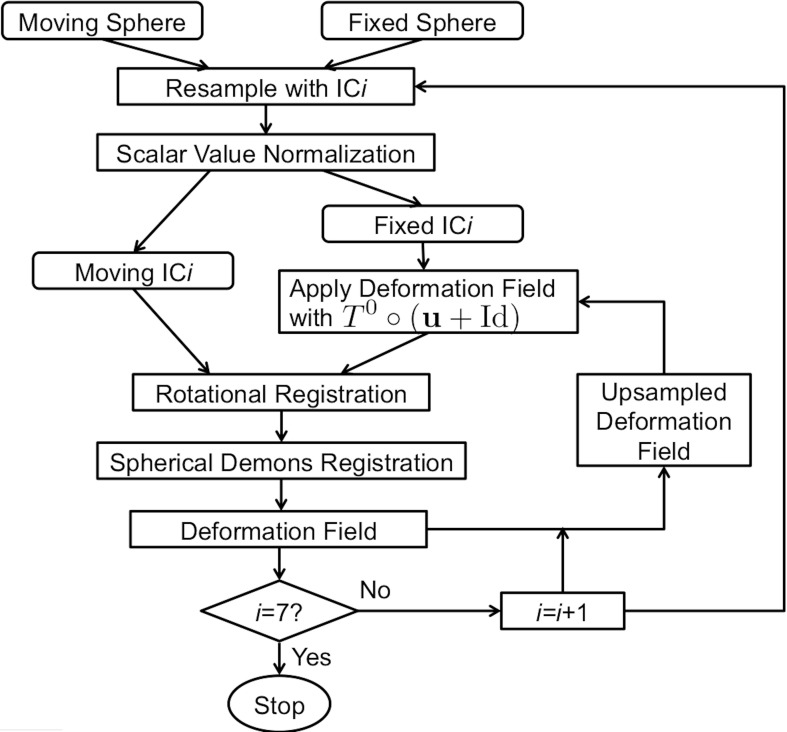
**The flowchart of the surface registration used to align surfaces for parcellation.** The inputs are spheres with geometry features and the output is the deformation field defined as vectors at vertices on the fixed sphere.

A flowchart of the overall registration algorithm is shown in Figure 2. The registration starts from the lowest resolution level IC4. To initialize each refinement level of registration, the deformation field from the previous level is used to warp the vertices on the original icosahedral mesh for the current level to their present location. A rotational transform based on the current scalar values is calculated followed by the diffeomorphic demons registration. The resulting rotation calculated at this level of registration is concatenated with the previous deformation and the process is repeated until all registration levels are completed. The rotational registration was added to each level since it was found that the diffeomorphic demons registration was trying to overcome a global rotation that resulted from changing the geometric features being used to drive the registration. Based on our initial evaluation of this rotational transform, we found that the rotation typically was approximately 1–2° and resulted in fewer iterations of the diffeomorphic demons registration for convergence. This is likely due to the fact that the rigid registration does not distort the shape of the triangles.

The rotational registration was performed by calculating a versor transform to minimize the difference between the fixed sphere and the moving sphere. The versor transform consisted of only rotations about the sphere center. The cost function was calculated using the mean squared metric between the normalized geometry features on the fixed sphere and the warped moving sphere. A gradient descent optimizer was used to search for the optimized rotation angles. This algorithm has several user tunable parameters, which are described in Table 2. The values of these parameters used in the current study are also included in the table. The value of the maximum step length shown in the Table 2 is defined for IC4, and is divided by 2(i − 1) where *i* is the current refinement level.

**Table 2:** **Parameters in rotational registration**.

**Parameter**	**Role**	**Value used**
Gradient magnitute tolerance	Stop the registration when the gradient magnitude is smaller than this value	1e-6
Maximum step length	Initial step length for the optimization	0.01
Minimum step length	Stop the registration when the current step length is smaller than this value	1e-9
Relaxation factor	Relax the current step length when gradient changes its direction	0.9
Number of iterations	Stop the registration after this number of iterations	30

(Yeo et al., 2010) extended the diffeomorphic demons registration from 3D images to 3D spheres with fixed radius, on which points are defined by 2D spherical coordinates.. The spherical registration uses the scalar values associated with vertices to bring the two surfaces into correspondence. By constraining the velocity field in tangent planes of the sphere, the exponential map of the velocity field transforms points to its local neighborhood on the sphere. In that way, the extended diffeomorphic demons algorithm maintains the topology of the sphere during registration. The constraint optimization problem (keeping the velocity vector in the tangent plane) was solved by introducing a local coordinate chart, which maps the tangent vector on the sphere *S*^2^ to the tangent vector at the origin of ℝ^2^. A deformation field smoothing technique was also utilized by Yeo et al. to perform regularization of the deformation field. Table 3 lists the parameters that can be adjusted for the spherical diffeomorphic demons registration. The value of σ needs to be adjusted based upon the shortest edge length, which is determined by the level of resolution, as the edge length becomes smaller when the resolution level of icosahedral mesh goes higher. The registration runs either for the specified number of iterations or until the similarity metric reaches a user defined convergence threshold.

**Table 3:** **Parameters in spherical diffeomorphic demons registration**.

**Parameter**	**Role**	**Value used**
ϵ	A non-zero positive coefficient	1/σ^2^
σ	Control the magnitude of the velocity field	$\sqrt{l}$
λ	Control the smoothing on the deformation field	1.0
Self regulated mode	Adjust ϵ and σ at each iteration	On
Minimum metric change	Stop the registration when metric changes smaller than this value	0.05
Number of iterations	Stop the registration after this number of iterations	500
Smoothing iterations	Number of Iterations to smooth the deformation field	40

l: the shortest edge length on surface.

### 2.6. Cortical parcellation schemes

For this study, two parcellation schemes were used to evaluate the automated cortical parcellation method employed. The first parcellation scheme was based on a manual parcellation of the cerebral cortex that was previously developed by our group (Crespo-Facorro et al., 1999, 2000b; Kim et al., 2000). This allowed us to compare the reliability as compared to a trained expert manual raters. The second parcellation scheme was based on FreeSurfer and allowed us to directly compare two automated methods for cortical parcellation.

**Figure 3:**
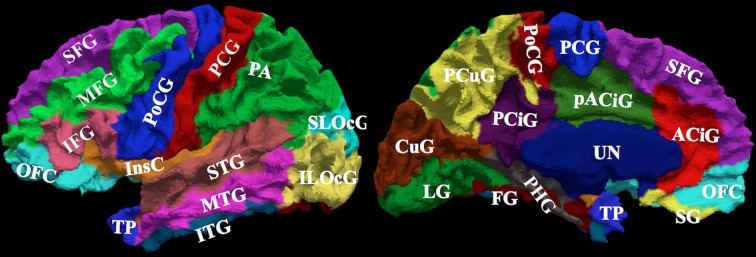
**The surface on the subject with the median overlap manually parcellated into 24 sub-regions.** The full name of each region is given in Table 4. The surface is shown in a lateral (left) and a medial (right) view.

Manual parcellation was performed on each hemisphere of the cerebral cortex by a trained and reliable anatomical rater. The parcellation of the cerebral cortex was performed by referencing the cortical surface, volumetric images, and anatomical landmarks. Each hemisphere of the cerebral cortex was divided into forty-one sub-regions as previously reported (Crespo-Facorro et al., 1999, 2000b; Kim et al., 2000). While manual parcellation remains the Ògold-standardÓ for evaluation of automated methods, it is an imperfect Ògold-standardÓ containing mislabeled regions at the individual subject level. The resulting parcellations were reviewed and regions split by reference planes (e.g., rostral, intermediate and caudal inferior temporal gyrus) were combined while a few small regions (e.g., Heschl's gyrus) were merged with surrounding regions. The motivation for the consolidation of regions was two fold. First, the reference planes could readily be defined after the initial parcellation to further subdivide the regions. Second, the manual parcellation of the cortex was time consuming and when applying to a large sample a number of inconsistent boundaries were identified upon secondary review especially related to smaller regions of interest. Given that we are dealing with an imperfect *gold standard*, some regions were consolidated to generate a more consistent parcellation across all subjects. Aside from the consolidation of regions, no additional attempt was made to refine the anatomical definitions even though errors in the anatomical definitions were noted in this review. The total number of regions was reduced to 24 regions per hemisphere [see Figure 3]. Table 4 summarizes the combination of regions *Automated Region* used in this study as compared to the previously reported guidelines *Manual Region*.

**Table 4:** **Cortical regions used in the automated parcellation**.

**Automated region**	**Abbrevation**	**Manual region**	**Lobar location**
Anterior cingulate gyrus	ACiG	r-ACiG	Frontal
Post anterior cingulate gyrus	pACiG	c-ACiG	Frontal
Inferior frontal gyrus	IFG	IFG	Frontal
Middle frontal gyrus	MFG	MFG	Frontal
Orbitofrontal Cortex	OFC	OFC, MFC	Frontal
Pre-central gyrus	PCG	PCG	Frontal
Superior frontal gyrus	SFG	HG, PP, PT, SFG, SMA	Frontal
Straight gyrus	SG	SG	Frontal
Fusiform	FG	FG, r-, c-OTG	Temporal
Inferior temporal gyrus	ITG	r-, i-, c-ITG	Temporal
Middle temporal gyrus	MTG	r-, i, c-MTG	Temporal
Parahippocampal gyrus	PHG	r-, c-PHG	Temporal
Superior temporal gyrus	STG	r-,c-STG	Temporal
Temporal pole	TP	TP	Temporal
Parietal	PA	r-, c-SMG, SPG, AG	Parietal
Posterior cingulate gyrus	PCiG	PCiG	Parietal
Pre-cuneus gyrus	PCuG	PCuG	Parietal
Post-central gyrus	PoCG	PoCG	Parietal
Cuneus	CuG	CuG	Occipital
Inferior lateral occipital gyrus	ILOcG	ILOcG	Occipital
Lingual	LG	LG	Occipital
Superior lateral occipital gyrus	SLOcG	SLOcG	Occipital
Insula	InsC	InsC	Frontaltemporal
Unlabeled	UN	SCA Subcortical structures	None

**Figure 4:**
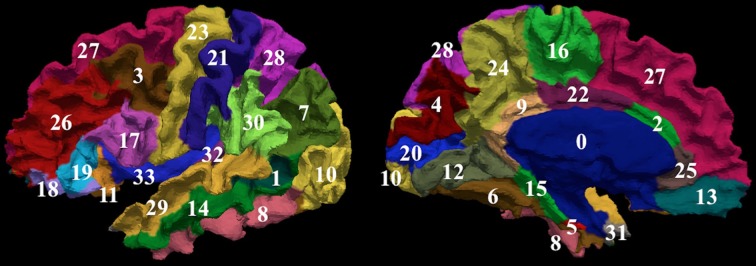
**The surface on a single subject parcellated into 34 sub-regions using FreeSurfer.** The regions as defined by FreeSurfer are provided in Table 5. The surface is shown with a lateral (left) and a medial (right) view.

To compare the proposed automated surface parcellation method with another commonly used surface analysis tool, the MRI scans from the fourty-nine subjects were also processed using FreeSurfer to label the cortical surface into 34 regions (33 cortical regions of interests and an unlabeled region) for each hemisphere as described by (Desikan et al., 2006). An example of the FreeSurfer cortical parcellation is shown in Figure 4. Table 5 lists regions of interest defined by FreeSurfer with the corresponding index used in Figure 4. It should be noted that the parcellation scheme developed by (Desikan et al., 2006) included a frontal pole region that was excluded from their reliability study. We have combined this region with the medial orbital frontal cortex region for this study.

**Table 5:** **FreeSurfer's regions**.

**Label**	**Region name**	**Label**	**Region name**
0	Unlabeled subcortical region	17	Pars opercularis
1	Banks superior temporal sulcus	18	Pars orbitalis
2	Caudal anterior cingulate cortex	19	Pars triangularis
3	Caudal middle frontal gyrus	20	Pericalcarine cortex
4	Cuneus cortex	21	Postcentral gyrus
5	Entorhinal cortex	22	Posterior-cingulate cortex
6	Fusiform gyrus	23	Precentral gyrus
7	Inferior parietal cortex	24	Precuneus cortex
8	Inferior temporal gyrus	25	Rostral anterior cingulate cortex
9	Isthmus-cingulate cortex	26	Rostral middle frontal gyrus
10	Lateral occipital cortex	27	Superior frontal gyrus
11	Lateral orbital frontal cortex	28	Superior parietal cortex
12	Lingual gyrus	29	Superior temporal gyrus
13	Medial orbital frontal cortex	30	Supramarginal gyrus
14	Middle temporal gyrus	31	Temporal pole
15	Parahippocampal gyrus	32	Transverse temporal cortex
16	Paracentral lobule	33	Insula

### 2.7. Surface atlas generation

**Figure 5:**
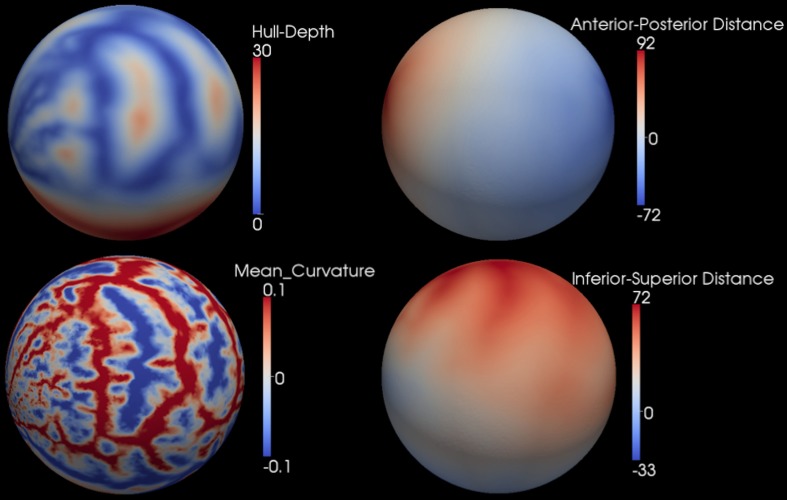
**The population average geometric features on the atlas sphere.** The feature types and scalar ranges are shown on the right side of each sphere.

The atlas representation was created by selecting one of the 35 training subjects as the template surface at random and then registering the remaining 34 subjects to this surface. The registration parameters used for this process are provided in Table 2 and Table 3. A separate atlas representation was generated for the right and left hemisphere. Once all of the surfaces were mapped onto the template surface, the average features were calculated on a per vertex basis. The average geometry features at each point in the spherical space were calculated. The resulting average geometry features are shown in Figure 5. The deformation field generated by aligning the training subject with the template was used to map manual labels from the individual subjects into the atlas space. After all of training subjects' labels were mapped into the atlas space, the label with the greatest probability was used to label each vertex. This step was performed separately for the manual and FreeSurfer labels resulting in two parcellation schemes defined on the atlas sphere. In addition, the atlas sphere contained the mean geometric features used to co-register the atlas onto each of the subjects in the testing set.

### 2.8. Surface parcellation

To generate the cortical parcellation based on either of the atlases described above, the anatomical T1 and T2 weighted images from the testing set described in Section 2.1. were analyzed using the BRAINS AutoWorkup procedure. The cortical surface was generated as described in Section 2.2.–Section 2.4. The surface registration on spheres was the same as used to generate the cortical atlas except that the atlas sphere was mapped onto the subject sphere. After the registration, the atlas-based labels were propagated from the atlas onto the subject surface. These fourteen subjects were used to assess the reliability of the automated parcellation by comparing the results defined either manually or with FreeSurfer.

### 2.9. Validity metrics

To quantitatively assess the reliability of the automated method, two metrics were used: DiceÕs coefficient and vertex accuracy. The Dice index was used to evaluate the similarity between the automated parcellation described here and the previous parcellation (either manual or FreeSurfer). The Dice index, *D* = 2(A ∩ B)/(|A| + |B|) was calculated based on surface area. A Dice coefficient of 0.0 corresponds to no overlap between the automated parcellation and the previous parcellation, while a Dice coefficient of 1.0 corresponds to identical regions of interest. The Dice coefficient was computed for each region in the testing set and mean and standard deviation reported.

Vertex accuracy provides the percentage agreement between the *gold-standard* label and the automated label for each vertex in atlas space. A vertex accuracy of 0 corresponds to no agreement between the automated and *gold-standard* labels at that vertex across the testing set, while a vertex accuracy of 100 corresponds to complete agreement across the testing set between the manual and automated labeling for the vertex. The vertex accuracy was summarized both using a histogram and visually on the atlas surface.

## 3. Results

### 3.1. Manual vs. automated labels

**Figure 6:**
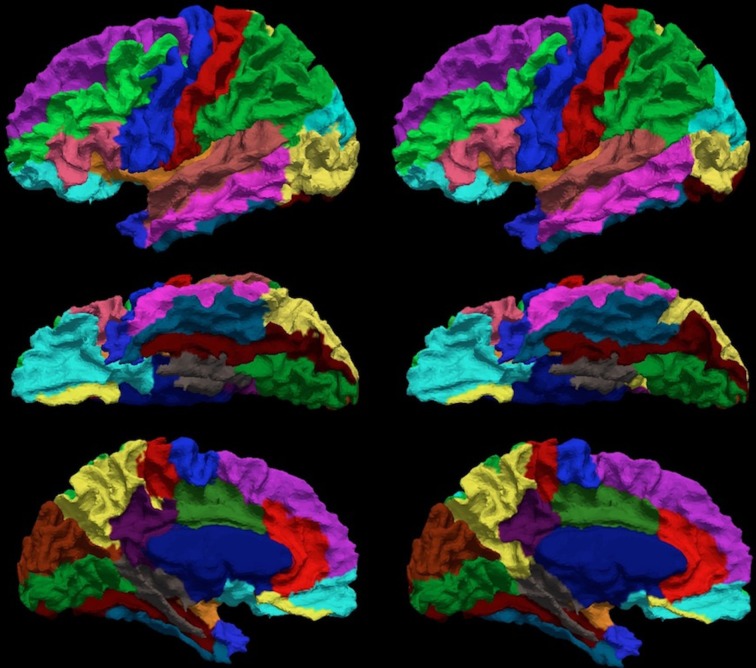
**The visual comparison of the manual (left column) and the automated (right column) parcellation for the subject shown in Figure 3.** The surface is parcellated into 24 sub-regions defined in Table 4. The subject is chosen by having the median Dice coefficient. The surface is shown in a lateral (top row), ventral (central row), and medial (bottom row) view.

The Dice coefficient between the automated and manual parcellation is shown in Table 6. The Dice coefficient ranged from 0.68 (fusiform gyrus) to 0.91 (insula and superior temporal gyrus). The mean Dice coefficient across all regions of the cortical surface was 0.84. Similar reliability was obtained for both the right and left hemisphere. The automated parcellation starting from the original DICOM images was completed in approximately 2 h of computer time and required no manual intervention. The quality of the resulting parcellation is shown in Figure 6. This figure shows the manual and the automated labels on a subject from the testing sample with the median Dice coefficient.

**Table 6:** **The Dice indices of regions**.

**Region**	**Left hemisphere**	**Right hemisphere**
ACiG	0.834 ± 0.060	0.816 ± 0.062
pACiG	0.877 ± 0.059	0.849 ± 0.034
IFG	0.846 ± 0.048	0.861 ± 0.040
MFG	0.884 ± 0.034	0.890 ± 0.030
OFC	0.900 ± 0.025	0.875 ± 0.040
PCG	0.900 ± 0.031	0.904 ± 0.030
SFG	0.888 ± 0.027	0.884 ± 0.032
SG	0.781 ± 0.113	0.812 ± 0.078
FG	0.713 ± 0.087	0.678 ± 0.112
ITG	0.749 ± 0.099	0.723 ± 0.107
MTG	0.824 ± 0.048	0.831 ± 0.045
PHG	0.733 ± 0.225	0.715 ± 0.126
STG	0.907 ± 0.025	0.910 ± 0.022
TP	0.835 ± 0.075	0.826 ± 0.053
PA	0.871 ± 0.033	0.880 ± 0.030
PCiG	0.784 ± 0.074	0.794 ± 0.054
PCuG	0.822 ± 0.043	0.845 ± 0.032
PoCG	0.879 ± 0.032	0.872 ± 0.038
CuG	0.843 ± 0.056	0.843 ± 0.046
ILOcG	0.759 ± 0.084	0.690 ± 0.109
LG	0.847 ± 0.057	0.847 ± 0.076
SLOcG	0.739 ± 0.070	0.736 ± 0.068
InsC	0.910 ± 0.016	0.894 ± 0.030
UN	0.931 ± 0.024	0.926 ± 0.024

Please refer to Table 4 for regions' details.

**Figure 7:**
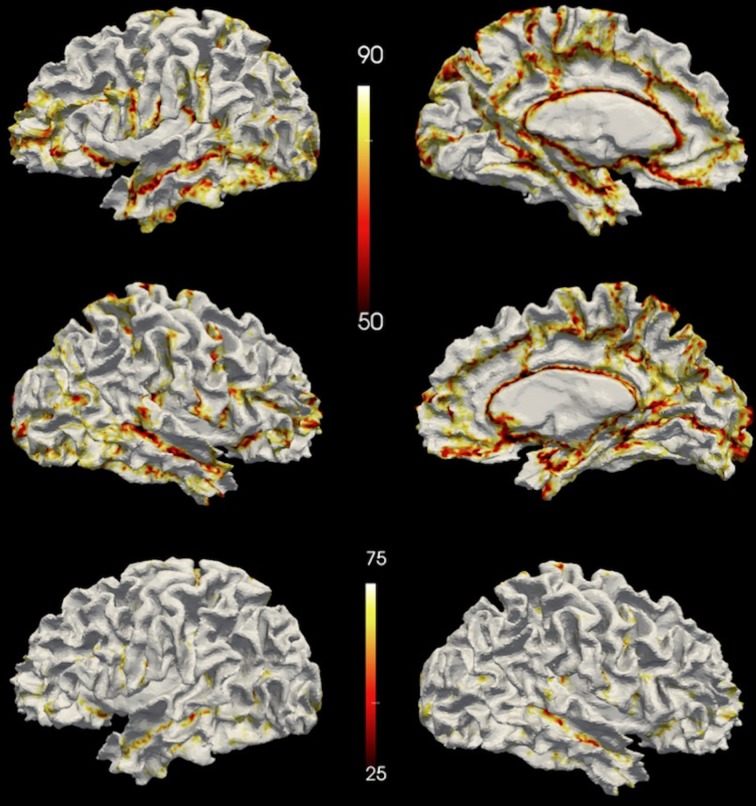
**The accuracy over the testing set visualized on a surface.** The top row shows accuracy on the left hemisphere and the central row shows accuracy on the right hemisphere. The bottom row shows the accuracy on the left hemisphere of the same subject as the top row but with a different scaling. Top two rows share the scale of 50~90%, while the bottom row uses the same scale (25%~75%) as being utilized in FreeSurfer's paper (Fischl et al., 2004).

**Figure 8:**
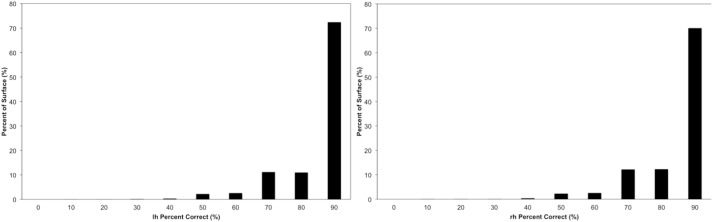
**The distribution of the accuracy in the atlas space.** Each bin is labeled by the minimal value of it. For example, the first bin “0” represents accuracy 0~10%, bin “10” represents accuracy 10%~20%, etc. The frequency is calculated by the number of vertices having accuracy represented by the bin.

The vertex accuracy in the atlas space was mapped onto the template surface for visualization (Figure 7). Approximately three quarters of the surface vertices were labeled correctly (with accuracy >90%). As expected, the locations with low accuracy were located along the borders between parcellated regions. In general, these regions with a large percentage of errors are thin bands between regions. Broader regions of uncertainty do exist and are often located where several regions intersect such as the cuneus, pre-cuneus, posterior cingulate, and unlabeled regions. Figure 8 shows the histogram for the vertex labeling accuracy. Approximately only 5% of the vertices were labeled with poor accuracy (< 60%).

### 3.2. Comparison with freesurfer

**Figure 9:**
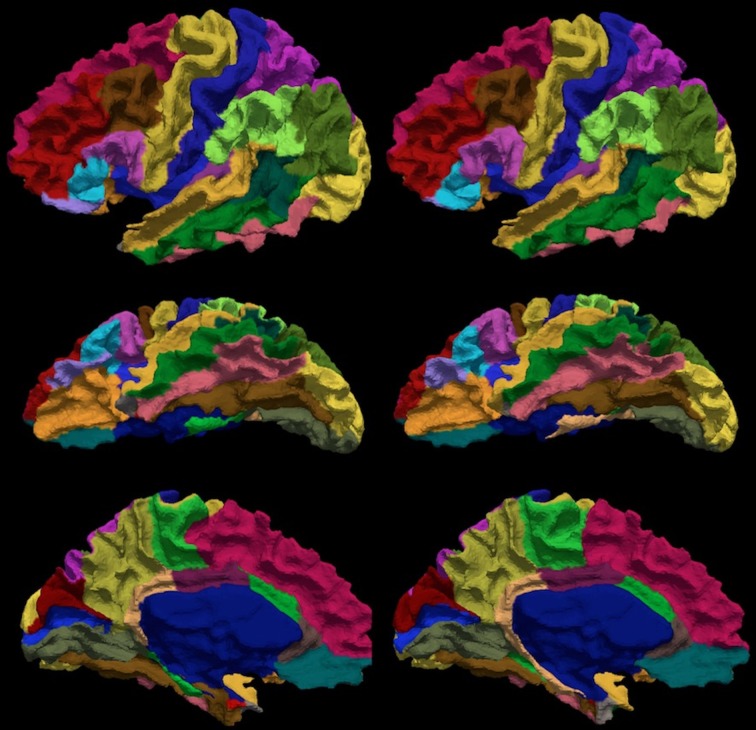
**The visual comparison of the FreeSurfer's (left) and the present (right) parcellation for a single subject.** The surface is parcellated into 34 sub-regions defined in Table 5. The subject is chosen by having the median Dice index of the similarity between the FreeSurfer's and the present pipeline's parcellation results. The surface is shown in a lateral (top), ventral (central), and medial (bottom) view.

The reliability of the automated parcellation pipeline proposed in this application was also compared against FreeSurfer using the Desikan atlas composed of 34 regions. The overall average Dice coefficient across 36 regions and fourteen testing subjects is 0.80, with a median of 0.86. Thirty-three out of thirty-six regions, which accounts for more than 99% of the whole cortical surface, were labeled with Dice coefficients greater than 0.70. The remaining three regions (entorhinal, temporalpole, parahippocampal) were small regions that account for less than 1% of the total cortical surface area. Figure 9 shows the labeled cortical surface using FreeSurfer and the proposed parcellation method.

## 4. Discussion

The Dice index evaluated the overlapping between two parcellations, automated vs. manual (*gold standard*) or automated vs. FreeSurfer. (Yeo et al., 2010) previously utilized the Dice coefficient to compare the spherical diffeomorphic demons registration to manual parcellation of cerebral cortex to compute the average Dice coefficient as 0.89 across regions and subjects. Using the automated pipeline proposed here, the Dice coefficient of 0.86 resulted from using the same evaluation was just slightly lower. In addition, we have presented the Dice coefficient for each region of interest averaged across subjects. This work was evaluated using a separate sample from that previously utilized by Yeo. Therefore, inter-subject variability in the cortical folding pattern could slightly influence the resulting reliability measures. For example, in a population of one hundred, there are 74 people that have a single post-central sulcus, and 26 having a double parallel pattern at the same location(Ono et al., 1990). In addition, secondary gyri and sulci are not always defined by geometry features used in aligning cortical surfaces together. Shallow sulci have small curvatures and hull depth that are not distinct across the cortex.

The Dice coefficient comparing the regions defined with the proposed method and FreeSurfer provides a measure of similarity between the two automated methods. Here, there is no Ògold standardÓ but the reliability of FreeSurfer has been previously published (Fischl et al., 2004; Desikan et al., 2006). The median Dice across the 36 regions for the testing set was 0.86. The vertex accuracy was previously reported by (Fischl et al., 2004) in the evaluation of FreeSurfer. To directly compare the method outlined in this paper to FreeSurfer, the same dynamic range for point accuracy as utilized by Fischl et al. was used to scale the results shown in Figure 7. Very few of the vertices (1.4%) were labeled incorrectly (with accuracy <25%). However in Figure 3 of Fischl et al. paper, a fairly significant number of vertices had an accuracy of lower than 25%. This would suggest that the method proposed in this paper is able to label the cortical surface with the same or better accuracy as compared to FreeSurfer. However, this was not directly tested here. As expected and evident in Figure 7, the largest errors all occur at the border between regions. The histogram in Figure 8 showed the distribution of vertex accuracy over the testing set. The distribution of accuracy was almost identical as for two hemispheres, and approximately 75% of the surface was labeled correctly (accuracy>90%). This is almost twice the percentage reported in FreeSurferÕs paper (Fischl et al., 2004). Even with high reliabilities (Crespo-Facorro et al., 1999, 2000b; Kim et al., 2000), it is still arbitrary to precisely define the borders in manual parcellation (Fischl et al., 2004). The true boundaries are based on cytoarchitecture and are not visible on conventional T1 and T2 weighted images similar to those utilized for this study. The average time for FreeSurfer to finish parcellating both hemispheres was approximately 20 h, while the automated parcellation proposed only needs 2 h on average. In the mean time, the proposed pipeline was developed with open source toolkits and is flexible to work with any parcellation scheme. The flexibility of our method allows any set of parcellation labels to be readily integrated into the pipeline by simply mapping the labels onto the surface atlas.

In this paper, we have developed a fully automated cortical labeling algorithm that is integrated into the BRAINS software. The resulting reliability was similar to FreeSurfer and could be completed in just a fraction of the CPU time. In this initial evaluation of the algorithm, no manual intervention was performed. Based on five regions having significantly different surface area measurements as compared to the manual definition (lingual gyrus, fusiform gyrus, inferior temporal gyrus, and posterior cingulate gyrus), the addition of additional anatomical information would likely significantly improve the results in these regions. For example, isthmus-cingulate cortex (label 9 in Figure 4) can go over to parahippocampal gyrus (label 15) in our parcellation as shown in Figure 9. It can be improved by locating the nearby structure, splenium of the corpus callosum and using it as the macroscopic ventral border as suggested by (Jones et al., 2006). We have created a general framework that allows any feature defined on the surface to drive the registration. In addition, standard VTK file formats are used allowing the user to readily define these scalar measures on the surface. For example, in future work, we are planning to couple automated landmark identification (Lu, 2010) as a preprocessing step. A manual rater would then be able to manually correct the location of the landmarks before using a spherical thin-plate spline to initialize the registration of the atlas with the subject.

### Conflict of interest statement

The authors declare that the research was conducted in the absence of any commercial or financial relationships that could be construed as a potential conflict of interest.
